# Clerical Health

**Published:** 1857-08

**Authors:** 


### CLERICAL HEALTH.

Henry Melvill is one of the eminent clergymen of London, and an old man; he looks like a stout, burly, beef-eating Englishman, of good height, broad shouldered, erect, full cheeks, and of a good color. It has been his habit to make one sermon a week, an exclusive labor, beginning early, and excluding all callers from his study many hours every day, writing it out in full, adding, erasing, transposing, modifying, until it is ready for being transcribed by a secretary, to be studied on Saturday, and delivered on Sunday.

In six years past he has preached and published two hundred and seventy-seven Tuesday lectures, not one of which is to be repeated; his published "sermons" make quite a library. Besides the lectures above, which admit of no substitutes, he is chaplain of the Tower, chaplain in ordinary to the Queen, and principal of a college where cadets are prepared for the East India Company's service. These offices involve a large amount of labor, and yield a large income. And this is the secret of a healthy, hard-working, and enduring old age: to be employed in a work which is our meat and drink, with a handsome compensation for the same; thus the worker is relieved of all care, all solicitude, of that eating, heart-shrivelling, brain-wasting, soul-destroying anxiety, which attends a high, honorable sense of pecuniary obligation.

A minister in debt, or stinted for means to supply his daily necessities, labors with a mountain weight upon him, and no wonder that, with an average pay of three hundred dollars a year, so many of them sink, in this country, long before their prime, into invalidism, if not into an early grave. "He studied too hard," is the verdict of the people; *He died of want*, is the verdict of truth—want of that liberal and sufficient support, which would enable them to labor with a cheerful heart and a singleness of purpose, which are essential to high success in any human calling.

We have seen lately, a statement that some seventeen Methodist churches were closed in a small district in New England. Of two hundred Baptist clergymen in Massachusetts, only twenty receive salaries exceeding three hundred and fifty dollars. And when it is remembered, that nine or ten years must be spent, with several thousand dollars in money, to qualify these men for their office, it is a burning shame, a living disgrace to church members of all denominations, that such a niggardly provision is made for those learned, talented, self-denying men, who are the salt of the earth, and without whose personal labors, in introducing the people into the knowledge of social, domestic, and civil duties, duties to each other, and duties to the state, as founded on Bible principles, this Democratic government of ours would go to pieces within any five years. We repeat it, as the dishonor of every law-abiding citizen, of every true lover of our Republican institutions, that the men to whose daily lives and labors, and weekly preaching, we owe so much, are permitted, in so many instances, to eke out a painful subsistence by resorting to various kinds of labor, that they may not become bankrupt at the end of any year. The hardest toil of all, for daily bread, is the toil of the brain. But to have to endure it, under the daily influence of skimpy food and clothing, is hard indeed. Well is it, that these men have something to feed upon, of which the *world* knows nothing, the hope of immortal bliss beyond, in the bosom of their Father, who is the King of all worlds.

